# Wafer-Scale Synthesis and Optical Characterization of InP Nanowire Arrays for Solar Cells

**DOI:** 10.1021/acs.nanolett.1c02542

**Published:** 2021-08-27

**Authors:** Lukas Hrachowina, Nicklas Anttu, Magnus T. Borgström

**Affiliations:** † NanoLund and Division of Solid State Physics, Lund University, Box 118, 221 00 Lund, Sweden; ‡ Physics, Faculty of Science and Engineering, Åbo Akademi University, FI-20500 Turku, Finland; § Department of Electronics and Nanoengineering, Aalto University, P.O. Box 13500, FI-00076 Aalto, Finland

**Keywords:** InP nanowires, MOVPE, PL, TRPL, reflectance, EBIC

## Abstract

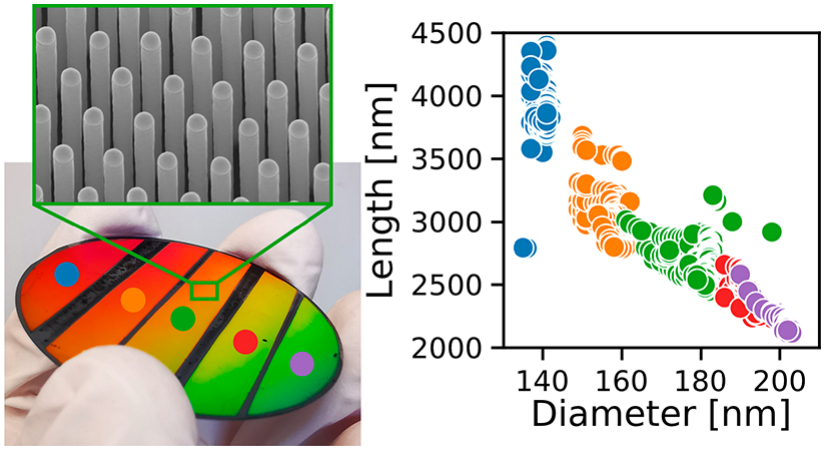

Nanowire solar cells have the potential to reach the same efficiencies as the world-record III–V solar cells while using a fraction of the material. For solar energy harvesting, large-area nanowire solar cells have to be processed. In this work, we demonstrate the synthesis of epitaxial InP nanowire arrays on a 2 inch wafer. We define five array areas with different nanowire diameters on the same wafer. We use a photoluminescence mapper to characterize the sample optically and compare it to a homogeneously exposed reference wafer. Both steady-state and time-resolved photoluminescence maps are used to study the material’s quality. From a mapping of reflectance spectra, we simultaneously extract the diameter and length of the nanowires over the full wafer. The extracted knowledge of large-scale nanowire synthesis will be crucial for the upscaling of nanowire-based solar cells, and the demonstrated wafer-scale characterization methods will be central for quality control during manufacturing.

## Introduction

Nanowires (NWs) are promising candidates for future solar cells.^1−3^ They hold the potential to reach the high efficiencies of world-record III–V semiconductor technology while using only about 10% of the material.^4,5^ Furthermore, NWs have unique advantages, such as the possibility to be integrated in polymers^6−9^ to create flexible solar cells with high efficiencies. Due to their geometry, NWs are radiation hard, which makes them ideal candidates for space applications.^10,11^ So far, the highest NW solar cell efficiencies have been reached by top-down processing,^12^ which does not take full advantage of the possibilities that NWs enable, and bottom-up synthesis of epitaxial NW arrays.^13,14^ Recently, there have been efforts to understand the efficiency-limiting factors of NW solar cells using electron-beam-induced current (EBIC) measurements—among other techniques such as cathodoluminescence,^15^ scanning photocurrent microscopy,^16^ and conductive-probe atomic force microscopy^17^—both on processed devices^18−21^ and on single NWs.^22−26^ However, to become a competitive photovoltaic technology, large-area NW solar cells are needed. Wafer-scale patterning necessary for NW synthesis has been shown by the use of nanoimprint lithography (NIL)^27^ and displacement Talbot lithography (DTL).^28^ Here, we demonstrate that wafer-scale synthesis of epitaxial InP NW arrays is feasible. In this work, we have used DTL to pattern 2 inch InP wafers. On one wafer, Sample 1, we defined five regions with different exposure doses to create NW arrays with different diameters. The other wafer, Sample 2, was patterned homogeneously. In order to characterize the material’s quality of both samples, we measured photoluminescence (PL) and time-resolved photoluminescence (TRPL) with a PL mapper. The most direct method to measure the length of epitaxial NWs is cross-sectional scanning electron microscopy (SEM) imaging, for which the sample has to be cleaved. Therefore, NW arrays are often imaged by use of a tilted stage, which increases the uncertainty in the measurement. In addition, only small areas with high magnification can be characterized by the use of SEM, and it would be too time-consuming to investigate a full wafer. *In situ* reflectivity is a convenient method to control the NW length during synthesis,^29^ but it gives information on a small central area of only about 1 mm^2^. By the creation of a simulated database that describes the reflectivity of NWs with a given diameter (*D*) and length (*L*), it is possible to extract those parameters from *ex situ* reflectivity measurements. This method has been used to measure the homogeneity across a small NW-array sample by time-consuming manual movement of the measurement spot over a 2 mm long line across the sample.^30^ Here, we use a PL mapper equipped with a white light source to automatically measure several thousand reflectivity spectra and utilize such a database extraction method to extract maps of the NW diameter and length over the full 2 inch wafer.

## Results and Discussion

**Figure 1:**
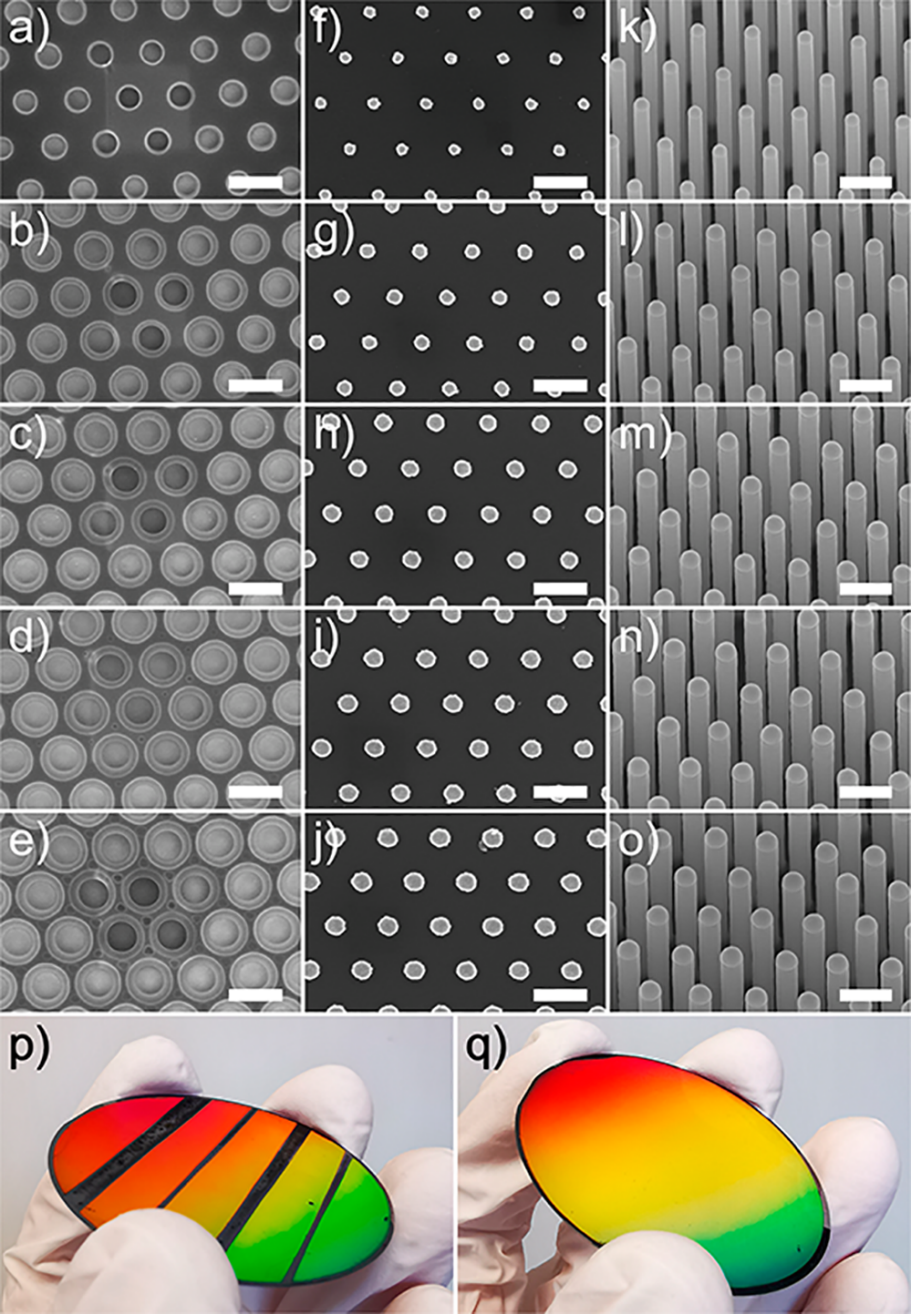
Representative SEM images of Sample 1: PMGI resist after development with the undercut visible as rings with bright contrast. The areas used for focusing appear darker and show enhanced contrast of the second-order diffraction (a–e). Au disks after evaporation and liftoff (f–j), and InP NWs tilted by 30° (k–o), with increasing DTL dose from 2.5 to 4.5 J/cm^2^ respectively. All scale bars are 500 nm long. Photographs of 2 inch InP wafers after NW synthesis: Sample 1 (p) and Sample 2 (q). The colors are due to visible light diffraction resulting from the periodicity of the NW arrays.

Figure 1 shows SEM images of the patterned wafer, Sample 1, after development of a polydimethylglutarimide (PMGI) resist (Figure 1a–e), Au disk evaporation and liftoff (Figure 1f–j), and NW synthesis (Figure 1k–o). The experimental methods are described in the Supporting Information. Furthermore, Figure 1p,q shows photographs of Sample 1 with five areas with different DTL doses and the homogeneously patterned Sample 2, respectively. The clearly visible angle-dependent coloring of the wafer can be attributed to the diffraction grating effect of the periodic NW arrays. The DTL dose was increased in steps of 0.5 mJ/cm^2^ from 2.5 to 4.5 J/cm^2^ from left to right.

The undercut that is created during development of the PMGI resist is dependent on the exposure time and is only 30 nm wide for the area with an exposure dose of 2.5 J/cm^2^ (Figure 1a), while adjacent undercuts in the area that was exposed with 4.5 J/cm^2^ almost merge together (Figure 1e). At 4.0 and 4.5 J/cm^2^ (Figure 1d,e) second-order diffraction effects can be observed between the intended exposures but these are not transferred to the substrate after gold evaporation and liftoff. The hole size after DTL exposure and development is difficult to measure because the resist is affected by the electron beam of the SEM. It is more precise to measure the diameter of the gold particles after evaporation and liftoff. However, for NWs synthesized by the vapor–liquid–solid (VLS) mechanism,^31^ it has to be considered that the NW diameter is determined by the interface area between the liquid seed particle and the substrate, which is dependent on the contact angle and the temperature-dependent solubility of the growth species in the seed particle.

In order to measure the homogeneity of the NW dimensions, we used a PL mapper equipped with a white light source and measured reflectance spectra of the NW arrays. Here, we performed the extraction of *D* and *L* similarly as in ref (30). That is, we created a database for modeled *R*(λ,*D*,*L*) for varying wavelength (λ), *D*, and *L* at a fixed period of 500 nm for the hexagonal array of nanowires. We varied *D* in the range from 0 to 500 nm in steps of 1 nm, *L* in the range from 0 to 10000 nm in steps of 1 nm, and λ_mod_ in the range from 400 to 750 nm in steps of 5 nm. We chose this upper limit for λ_mod_, since at longer wavelengths the reflectance from the metal particle becomes more crucial for the optical response of the array. The lower limit of 400 nm is given by the lower limit of the measurements. Due to conveniently available tabulated refractive index values,^32^ we modeled the metal as pure Au, whereas in reality it has formed a Au–In alloy. For the refractive index of InP, we used values from Glembocki and Piller.^33^

The modeling was done using the Fourier modal method,^34^ and the Au particle was assumed to be a hemisphere on top of the InP nanowires, which was modeled having a circular cross section. In the modeling, we assumed that the light is incident at a normal angle, since the measurements were performed with an objective of small numerical aperture (NA)*.*

**Figure 2:**
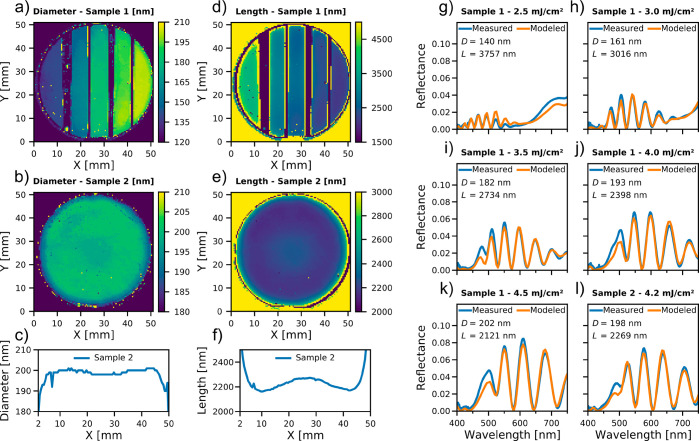
Extracted *D* (a, b) and *L* (d, e) from the measured *R*_exp_(λ) for Sample 1 (a, d) and Sample 2 (b, e). Line scans of extracted *D* (c) and *L* (f) through the center of Sample 2. (g–l) *R*_exp_(λ) at the center of the regions with different DTL exposure doses on Sample 1 and the center of Sample 2. We also show here the best-matching *R*_mod_(λ,*D*,*L*) values for the *D* and *L* values that minimize *s*(*D*,*L*). These extracted *D* and *L* values are stated within each subfigure.

For the extraction of *D* and *L* from the measured spectrum *R*_exp_(λ) at a given position on the wafer, we calculated *s*(*D*,*L*) = ∑_*i*_(*R*_exp_(λ_*i*_) – *R*_mod_(λ_*i*_, *D*,*L*))^2^, the sum of the square deviations between measured and modeled data, with λ_*i*_ being the modeled wavelengths. In this calculation of *s*(*D*,*L*), we smoothed *R*_exp_ over five measurement points, that is, over 2.8 nm in wavelength, and interpolated the smoothed *R*_exp_ onto the modeled wavelengths λ_*i*_. We assigned those values that minimize this *s*(*D*,*L*) as extracted *D* and *L*. The model accurately fits the data obtained by mapping Samples 1 and 2, including the five separate areas on Sample 1 (Figure 2).

For Sample 1 we observe, as expected, a continuously increasing extracted *D* for increased DTL exposure doses (Figure 2a) and, similarly, a decreasing extracted *L* (Figure 2d). This wafer mapping demonstrates both homogeneous *D* and *L* within each of these five areas.

Figure 2b,e shows the extracted *D* and *L* maps of Sample 2, respectively. From a line scan through the center of the wafer, we find a homogeneous *D* of 200 nm, except for the last 2 mm at the edge of the wafer, where the diameter drops by 10 nm (Figure 2c). In contrast, *L* peaks at the center of the wafer with a value of 2270 nm and drops to 2160 nm at approximately 15 mm from the center, after which it starts increasing rapidly toward the edge of the wafer (Figure 2 f). Similar gradients in *L* were confirmed by SEM measurements (not shown).

In Figure 2g–l we show the measured and best-matching modeled reflectances for the different DTL exposure doses of Sample 1 and the center of Sample 2. For all these points, we find that *R*_exp_ and *R*_mod_ corresponded well to each other in both spectral shape as well as in absolute value. Furthermore, the spectra between different positions vary noticeably, depending on both *D* and *L*, which allows for the simultaneous extraction of *D* and *L*.^30^

**Figure 3:**
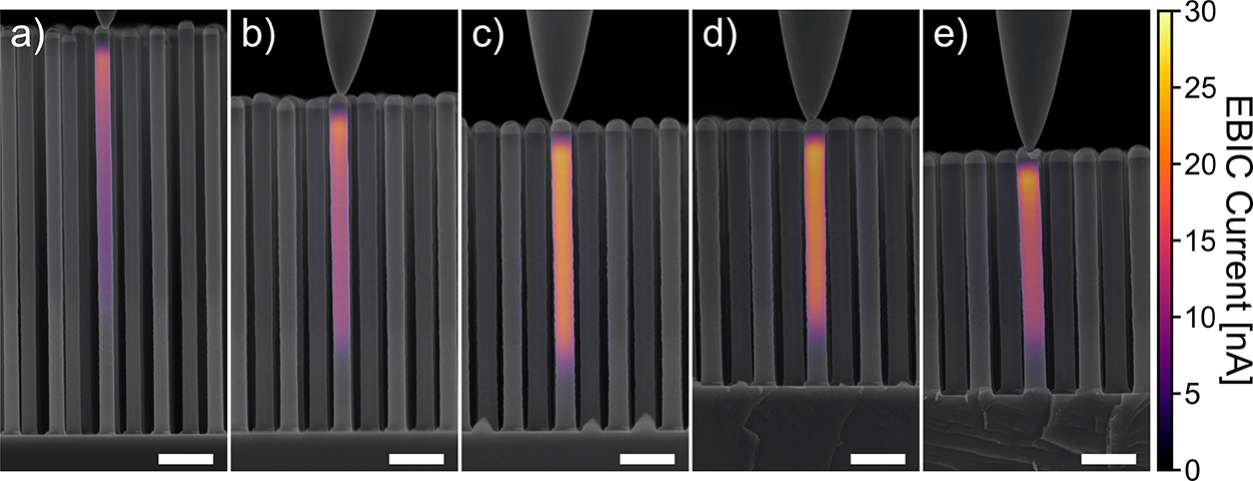
Cross-sectional SEM and superimposed EBIC measurements of Sample 1 with DTL exposures of 2.5 mJ/cm^2^ (a), 3.0 mJ/cm^2^ (b), 3.5 mJ/cm^2^ (c), 4.0 mJ/cm^2^ (d), and 4.5 mJ/cm^2^ (e). All scale bars are 500 nm long.

In order to verify the lengths and diameters obtained from the above optical extraction, we characterized the nanowire length at the cross section of Sample 1 using SEM. Simultaneously, we measured the EBIC currents of at least five NWs from each of the different regions. The growth conditions had been optimized for 9 × 11 mm^2^ substrates and an NW diameter of 200 nm. As seen from the EBIC measurements (Figure 3), the doping incorporation was not affected strongly by the substrate size or by the NW diameter. All across Sample 1 similar EBIC profiles were measured, although the highest currents were measured for 3.5 and 4.0 J/cm^2^. This proves that all measured NWs, regardless of *D* and *L*, have a well-defined p–i–n junction suitable for further processing to NW solar cells.

Table 1 gives *D* and *L* values of Sample 1 both extracted from reflectance maps (Figure 2g–k) and measured in the SEM: that is, from Figure 3. We find excellent agreement between the two measuring methods. The cross-sectional SEM was performed on a central horizontal line of the wafer, using several NWs of five different vertical positions. For the optical extraction, on the other hand, we analyzed the whole areas of the regions with different DTL exposures, excluding the outermost 1 mm. In Figure S1 we plot the extracted *L* against the extracted *D* on the basis of these 2939 measurement points. Note that we did not cleave the full wafer Sample 2 for accurate side-view SEM measurements, since it was used for later processing. Instead, on the basis of the verification of the extraction of *D* and *L* on Sample 1 with the optical method, we rely on this optical extraction for Sample 2.

**Table 1:** Comparison of *D* and *L* Extracted from the Reflectance Measurements and Cross-Sectional SEM Measurements of Each of the Five Areas of Sample 1^a^

DTL dose (mJ/cm^2^)	*D* (nm) from *R*_exp_	*L* (nm) from *R*_exp_	*D* (nm) from SEM	*L* (nm) from SEM
2.5	140.3 ± 6.4	3814 ± 271	134 ± 5	3580 ± 96
3.0	157.8 ± 3.3	3028 ± 139	164 ± 5	2958 ± 27
3.5	179.9 ± 3.7	2665 ± 267	182 ± 4	2707 ± 15
4.0	193.5 ± 3.2	2367 ± 133	196 ± 3	2412 ± 13
4.5	201.6 ± 1.6	2167 ± 57	199 ± 4	2089 ± 16

a See Figure 2g–l for the corresponding reflectance spectra.

**Figure 4:**
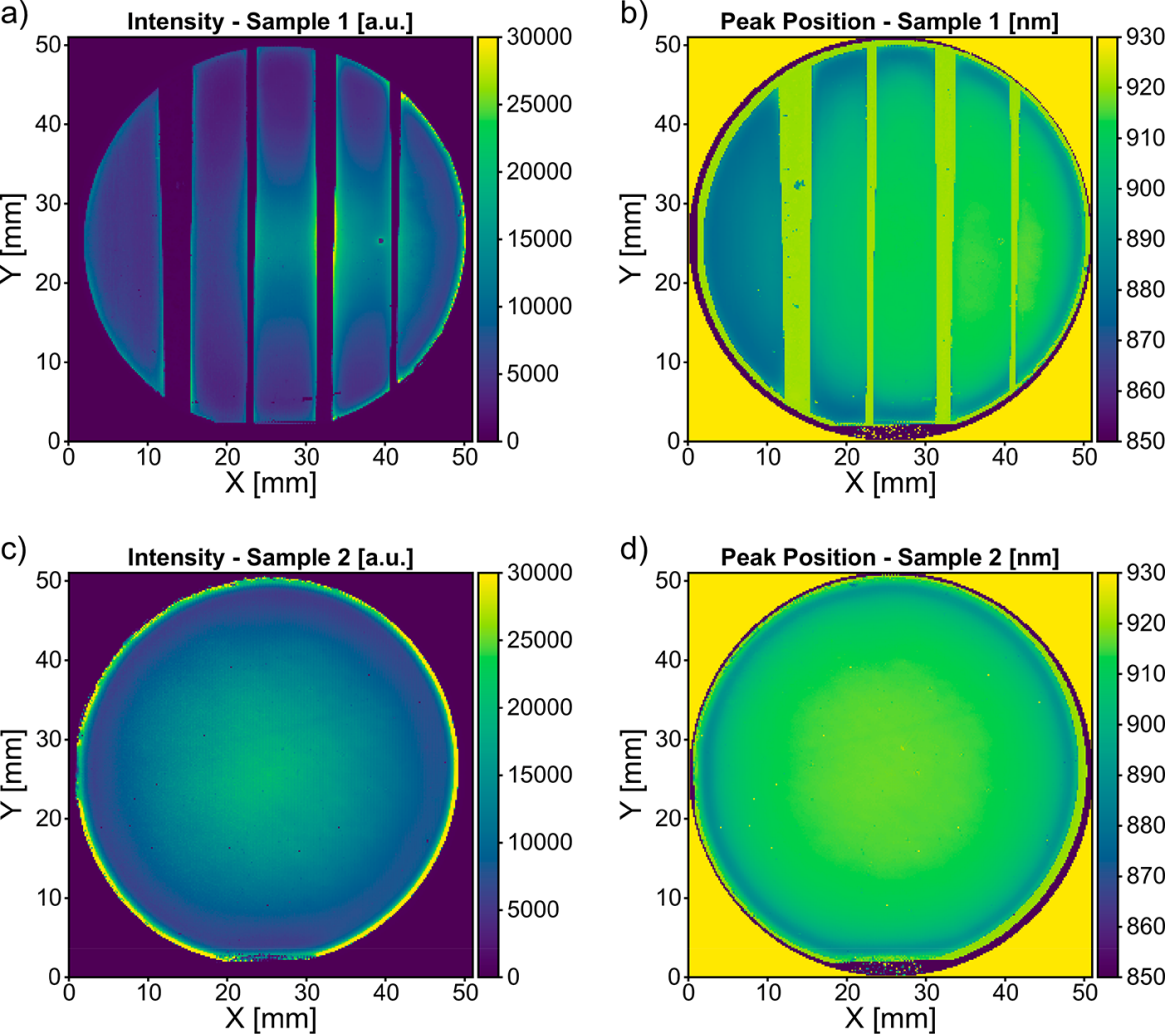
Photoluminescence maps of wafers with varied (a, b) and homogeneous (c, d) DTL doses. (a) and (c) show the highest measured intensity of every pixel. (b) and (d) show the peak position of the measured PL.

In order to measure the homogeneity of the material’s quality, we measured PL spectra over the samples in steps of 0.2 mm. The peak maxima and positions of the resulting PL maps are shown in Figure 4. Sample 1 demonstrates two effects. First, the PL intensity shows radial symmetry and peaks at the center of the wafer. Second, we notice an increased intensity not only toward the edge of the wafer but also toward the edges of the lithography-defined areas. Both effects complicate a direct comparison between the different areas, but the areas with higher DTL doses show higher PL intensities in comparison to the areas with lower DTL doses. Sample 2 exhibits a similar intensity distribution, but without the additional edge effect from lithography.

The peak position does not show such a pronounced radial dependence and is more homogeneous over the area of the wafer. Toward the edge of the wafer, but not toward lithography-defined edges, we measure a higher band gap. To explain this band gap variation, we note that InP NWs are known to be polymorphic and occur in both zincblende and wurtzite crystal structures, with band gaps at room temperature corresponding to 1.35 and 1.42 eV, respectively:^35^ that is, 918 and 873 nm in corresponding photon wavelength. Furthermore, the top n segment tends to be more wurtzite than p-doped InP nanowires, for which it has been shown that Zn doping leads to a zincblende structure.^36^ Additionally, most of the light absorption occurs at the tip of the NWs.^37^ Therefore, we explain the blue-shifted peak position toward the edge of the wafer with an increase in NW length toward the edge, which is expected to increase the length of the n segment as well. Thus we expect, due to the longer n segment, relatively stronger absorption in the n segment than in the p segment toward the edge of the wafer and therefore relatively stronger wurtzite-type, blue-shifted PL at the edge of the wafer.

Additionally, Sample 1 shows that the blue shift in band gap is dependent on the NW diameter and is more prominent for thinner NWs. As the reflectance measurements only yield the total NW lengths, we used the EBIC measurements to estimate the lengths of the p, i, and n segments at the center of each region, as shown in Figure S2. The n segments of the NWs with the thinnest diameter are slightly longer than the n segments of the other NWs, but not long enough to explain the blue shift of the whole region. However, we note that a *D* value of 140 nm is actually optimized to absorb wavelengths from 600 to 650 nm.^38^ Therefore, we modeled the diameter-dependent axial absorption profiles of the 632 nm excitation laser, shown in Figure S3. Using the estimated segment lengths from the EBIC measurements, we calculated the absorption of the different segments of the p–i–n NWs. Due to the strong absorption of the laser wavelength, the relative absorption in the n segment in comparison to the p segment, for NWs with a *D* value of 140 nm, is about 100 times higher than for the thicker NWs, as can be seen in Table 2. Thus, for the smallest diameter region, we expect a much stronger relative photogeneration into the n segment in comparison to the p segment and hence more pronounced wurtzite type, blue-shifted photoluminescence.

**Table 2:** Modeled Absorption *A* of the 632 nm Laser for Different *D* Values in the Five Regions of Sample 1, with Estimated Segment Lengths Based on the EBIC Profiles in Figure S2

	*D* (nm)				
	140	161	182	193	202
*A*_Au-particle_ (%)	2.97	2.15	7.41	4.95	6.49
*A*_n segment_ (%)	32.8	17.2	16.8	16.1	13.3
*A*_i segment_ (%)	63.0	65.8	49.8	47.4	41.8
*A*_p segment_ (%)	0.25	10.1	14.2	15.9	16.8
*A*_n segment_/*A*_p segment_	132	1.70	1.18	1.01	0.796

**Figure 5:**
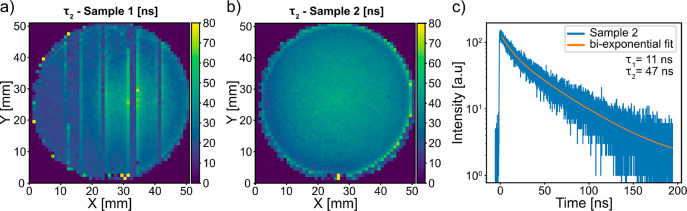
Charge carrier lifetime maps of the TRPL kinetics of Sample 1 (a) and Sample 2 (b). Representative TRPL kinetic from the center of Sample 2 (c).

Furthermore, we have measured TRPL maps of both samples. Due to the increased measurement time, a larger step size of 1 mm was used to give a feasible time for the full mapping. Also with this larger step, the different areas of Sample 1 are clearly distinguishable. We used a biexponential decay to fit the TRPL kinetics. The decay curves consist of a fast initial decay with a short charge carrier lifetime τ_1_ followed by a second decay with a longer charge carrier lifetime τ_2_. We did not observe significant differences over the sample area in the first initial decay. In order to illustrate the TRPL maps, Figure 5 shows the second, longer, charge carrier lifetime τ_2_ for every measured decay curve.

The decay times of the TRPL kinetics show a trend similar to that in the PL intensity maps, with a longer τ_2_ correlating with a higher PL intensity. Thus, (i) the carrier lifetime shows radial dependence that peaks toward the center of the wafer, (ii) the carrier lifetime increases toward the edges on both samples, and (iii) although the radial gradient dominates on Sample 1, we notice that the NWs with the largest diameter have a longer carrier lifetime than the NWs with the smallest diameter. Astonishingly, we measure carrier lifetimes that are 10 times longer than those of similar NW arrays with a sample size of 9 × 11 mm^2^ that were homogeneously doped instead of having p–i–n junctions.^26^ Long carrier lifetimes have been reported in mixed-phase InP NWs,^39,40^ and it has been shown that the type II band offset between zincblende and wurtzite InP can increase recombination lifetimes.^41^ However, we have studied similar NWs using transmission electron microscopy and observed mixed crystal phases even for homogeneously doped NWs;^42^ consequently, it is not obvious that the type II band offset is the cause for the increase in the carrier lifetimes of the NWs discussed in this paper. Another beneficial factor for the lifetime is definitely the size of these samples, as the center of samples that are 9 × 11 mm^2^ in size corresponds to an edge distance of 5 mm on the 2 inch wafer and we see here that τ_2_ increases continuously toward the center of the 2 inch wafer. However, just such a sample-size effect does not explain the full increase in carrier lifetimes, and therefore the p–i–n junction clearly increases the carrier lifetime, presumably by separating charge carriers along the NW axis. This is expected to increase the carrier lifetime, since the separated excess carriers can then recombine only by diffusing back to the junction region with a very low concentration of electrons and holes. A quantitative analysis of the recombination in TRPL experiments has been shown with three-dimensional numerical simulations,^43^ but the model has yet to be extended to take p–i–n junctions into account.

## Conclusion

In conclusion, we have synthesized InP NW arrays on 2 inch wafers, which is crucial to fabricate solar cells with areas on the order of cm^2^, characterized them by optical methods, and compared the results to modeling results. We have studied the homogeneity of the material’s quality across the wafer by measuring PL and TRPL maps. Furthermore, the NW diameter and length could be obtained by fitting reflectivity spectra to a calculated database. Cross-sectional SEM showed an excellent agreement with the dimensions obtained by these optical measurements. Finally, we measured EBIC on NWs from different areas of the wafers and showed that the dopants are homogeneously incorporated. This work paves the way for the fabrication of NW solar cells with an area of up to 2 inch wafers, and the methods we have used are applicable to 4 inch or even larger wafers.

## Supporting Information Available

Experimental methods, statistics of optically extracted NW dimensions of Sample 1, extracted EBIC profiles of Sample 1, and diameter-dependent axial absorption profiles for the NWs from Sample 1 (PDF).
