# Supplementary material for: Wafer-Scale Synthesis and Optical Characterization of InP Nanowire Arrays for Solar Cells
Source: Nano Lett. 2021 Aug 27;21(17):7347–53. doi: 10.1021/acs.nanolett.1c02542 (PMC8431724; doi:10.1021/acs.nanolett.1c02542)
Supplement: Supplementary file 1 — nl1c02542_si_001.pdf [file nl1c02542_si_001.pdf]

Supporting Information for

Wafer-scale Synthesis and Optical Characterization of InP Nanowire Arrays for Solar Cells

Lukas Hrachowina[1], Nicklas Anttu[2,3] and Magnus T. Borgström[1,]*

[1] NanoLund and Division of Solid State Physics, Lund University, Box 118, 221 00 Lund, Sweden

[2] Physics, Faculty of Science and Engineering, Åbo Akademi University, FI-20500 Turku, Finland

[3] Department of Electronics and Nanoengineering, Aalto University, P.O. Box 13500, FI-00076 Aalto, Finland

[*]Email: magnus.borgstrom@ftf.lth.se

KEYWORDS. InP nanowires, MOVPE, PL, TRPL, reflectance, EBIC

EXPERIMENTAL METHODS.

DTL The InP(111)B wafers were pre-baked for 10 min at 200 °C on a hotplate to improve the adhesion of the resists. Then, PMGI SF3S was spin coated at 2000 rpm for 45 s and baked for 10 min at 200 °C, after which PAR1085S90 was spin coated at 4500 rpm for 45 s and baked for 1 min at 90 °C. The wafers were then exposed to a 193 nm ArF excimer laser within a PhableR 100 DUV photolithography system to define a hexagonal array with a period of 500 nm. In order to define regions with different exposure doses, black plastic stripes were placed on the phase shift mask to block the UV light. After the exposure, the wafers were baked for 50 s at 100 °C and then developed for 60 s in MF24A. 65 nm of gold was deposited onto the wafers with a Temescal e-beam evaporator. Excessive resist with gold on top was removed by use of Remover 1165, and subsequently the wafers were cleaned with Millipore cleaned water.

MOVPE The InP NWs were synthesized in a laminar flow Aixtron 200/4 MOVPE reactor. During all synthesis steps a reactor pressure of 100 mbar and a total flow rate of 13 l/min were used. The parameters for NW synthesis were based on 9×11 mm^2 substrates as described in Ref[1]. A molar fraction of trimethylindium (TMIn) of $\chi_{TMIn} = 8.91\times10^{-5}$, and of phosphine ($PH_3$) of $\chi_{PH3} = 6.92\times10^{-3}$ were introduced into the reactor for a low-temperature pre-annealing nucleation step to preserve the periodic gold pattern[2]. Then, the substrate was annealed at 550 °C under PH_3 atmosphere ($\chi_{PH3} = 3.46\times10^{-2}$) to remove surface oxides. The reactor temperature was reduced to 440°C, and to start the NW synthesis, TMIn was introduced into the reactor ($\chi_{TMIn} = 5.94\times10^{-5}$) and the PH_3 flow was reduced ($\chi_{PH3} = 6.92\times10^{-3}$). At the same time, hydrogen chloride (HCl) was introduced to prevent tapering of the NWs *in-situ* ($\chi_{HCl} = 1.23\times10^{-4}$)[3]. Diethylzinc (DEZn) and tetraethyltin (TESn) were used for p- and n-doping respectively ($\chi_{Zn} = 1.11\times10^{-5}$, $\chi_{Sn} = 4.29\times10^{-5}$). In order to compensate for the n-type background doping of nominally intrinsic InP, a DEZn molar

fraction of $\chi_{DEZn} = 0.3\times10^{-7}$ was used in the middle segment of the p–i–n junction[1]. The NW length was monitored *in-situ* by using reflectometry and the deposition time was increased to compensate for the decreased growth rate caused by the larger substrate area.

PL mapper A PL mapper from Enlitech was used to measure PL, TRPL and reflectance. PL maps were measured with a 632 nm laser diode with an optical power of 3.8 mW in steps of 0.2 mm. A 700 nm low-pass filter was used to block reflection of the laser. The TRPL decays were measured by the use of a time-resolved single photon counter and a pulsed 632 nm laser diode with an optical power of 6 μW and a repetition frequency of 5 MHz. A step size of 1 mm was used, and every position was measured for 10 s. For both PL and TRPL, a 20× objective with a numerical aperture (*NA*) of 0.40 and a spot size of 80 μm was used for an optimal signal to noise ratio. The reflectance spectra were measured with a white light source from 400 nm to 1200 nm with a wavelength (λ) resolution of 0.57 nm. Note that the optical model for creating the simulated reflectivity database assumes, for convenience of the numerical calculations, parallel light incidence. Therefore, a 5× objective with a *NA* of 0.15, that is, smaller than in the PL and TRPL measurements, was used in these reflectance measurements. A metal mirror was used as a reference.

SEM and EBIC A Zeiss LEO 1560 thermal field emission SEM was used for top-view and tilted imaging. Cross-sectional SEM and EBIC were measured in a Hitachi SU8010 SEM equipped with a Point Electronic EBIC amplifier. The samples were cleaved and glued on 90° stubs. Single NWs were contacted by use of a tungsten probe equipped to a Prober Shuttle from Kleindiek Nanotechnik. At least five NWs were measured in every region of Sample 1. An acceleration voltage of 5 kV and a beam current in the order of tens of pA were used for all measurements.

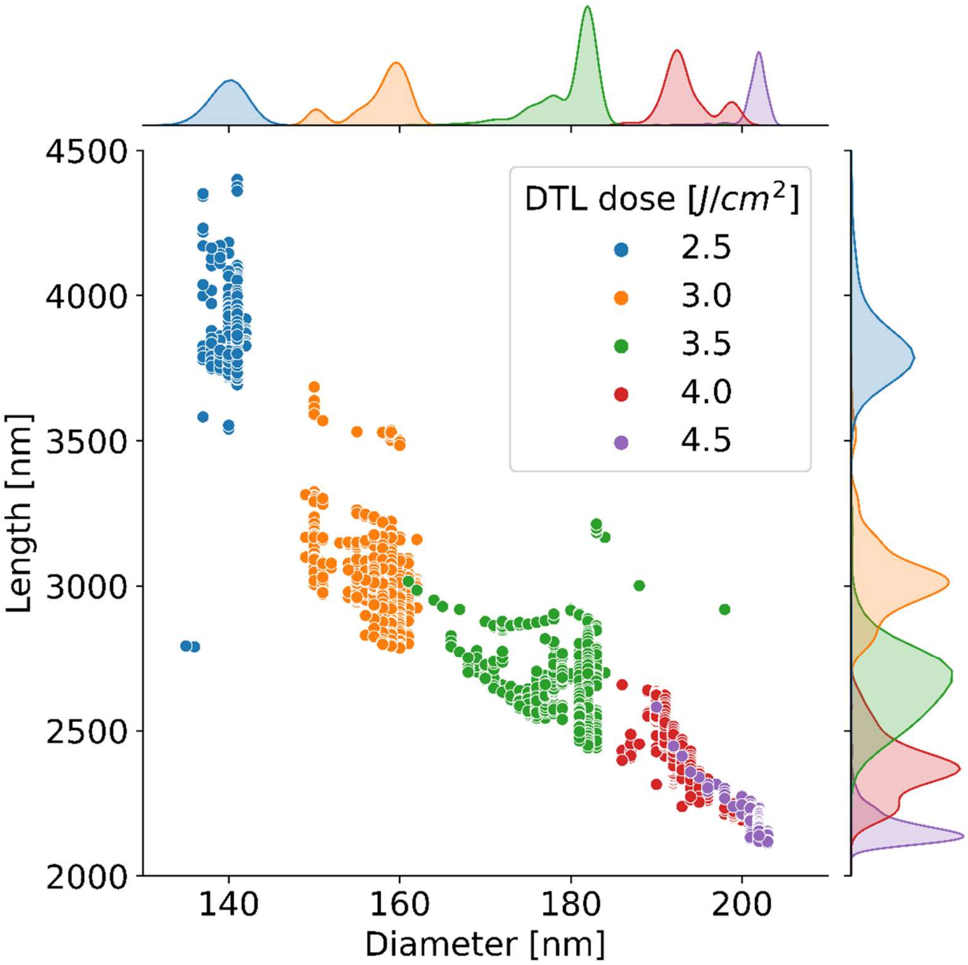

Figure S1: Extracted Lengths vs extracted Diameters of the different DTL exposures of Sample 1. The extraction is based on rectangular selection areas for each DTL exposure region, excluding the outermost 1 mm, resulting in 115, 156, 228.25, 162, and 73.5 mm^2, based on 460, 624, 913, 648, and 294 measured R spectra, respectively.

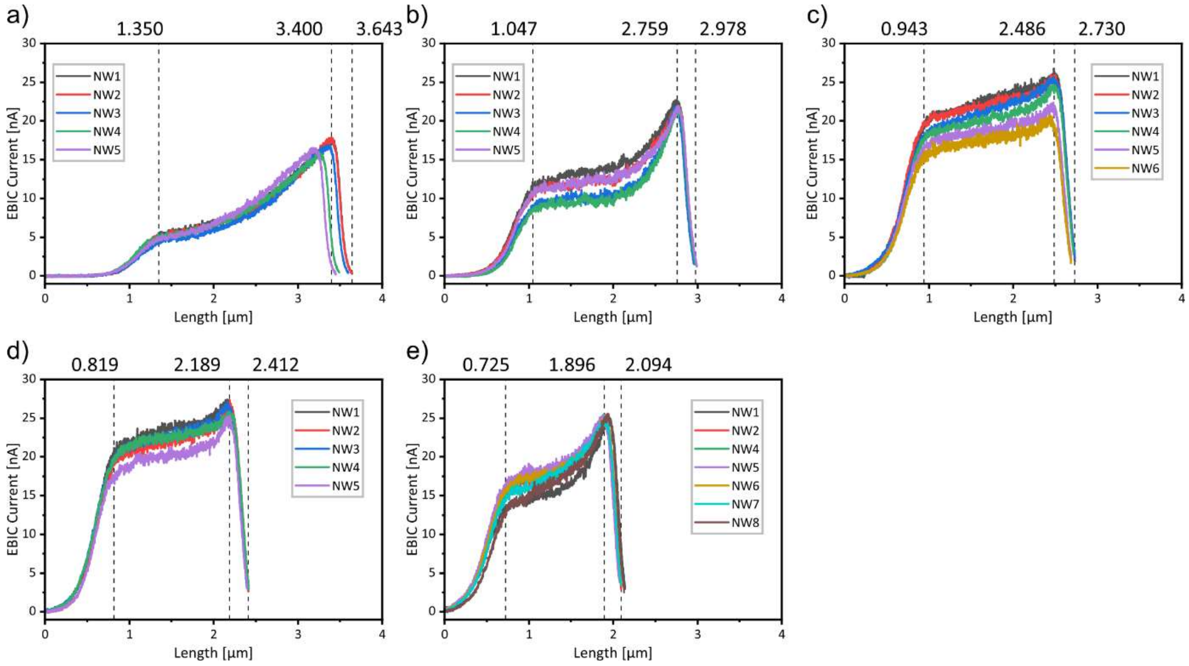

Figure S2: Extracted EBIC profiles of Sample 1. a-e corresponds to DTL exposures from 2.5 J/cm² to 4.5 J/cm² in steps of 0.5 J/cm². At least five NWs were measured for every DTL exposure dose. The vertical dashed lines indicate the interfaces between p-, i-, and n-segments and are used to estimate the segment lengths.

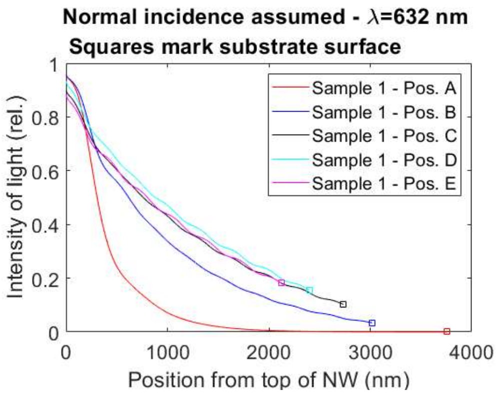

Figure S3: Diameter dependent axial absorption profiles for the NWs from Sample 1. Here, normal incident light was modeled, since only minor effects in absorption are expected from the varying incidence angles within the moderately small NA of 0.4 used in the experiments[4], corresponding to maximum incidence angle of just 24°. The top of the NW is defined as the interface between the InP NW and the Au particle. The positions A-E correspond to a-e in Figure S2.

REFERENCES

1. Otnes, G., et al., *Understanding InP Nanowire Array Solar Cell Performance by Nanoprobe-Enabled Single Nanowire Measurements.* Nano Lett, 2018. **18**(5): p. 3038-3046.
2. Otnes, G., et al., *Strategies to Obtain Pattern Fidelity in Nanowire Growth from Large-Area Surfaces Patterned Using Nanoimprint Lithography.* Nano Res., 2016. **9**(10): p. 2852-2861.
3. Borgström, M.T., et al., *In Situ Etching for Total Control over Axial and Radial Nanowire Growth.* Nano Res., 2010. **3**(4): p. 264-270.
4. Ghahfarokhi, O.M., et al., *Performance of GaAs Nanowire Array Solar Cells for Varying Incidence Angles.* IEEE J. Photovolt., 2016. **6**(6): p. 1502-1508.
